# Imaging far and wide

**DOI:** 10.7554/eLife.21072

**Published:** 2016-09-23

**Authors:** Raghav K Chhetri, Philipp J Keller

**Affiliations:** HHMI Janelia Research Campus, Ashburn, United Stateschhetrir@janelia.hhmi.org; HHMI Janelia Research Campus, Ashburn, United Stateskellerp@janelia.hhmi.org

**Keywords:** confocal microscopy, embryology, organogenesis, microscopy, Mouse, Rat

## Abstract

A custom-built objective lens called the Mesolens allows relatively large biological specimens to be imaged with cellular resolution.

**Related research article** McConnell G, Trägårdh J, Amor R, Dempster J, Reid E, Amos WB. 2016. A novel optical microscope for imaging large embryos and tissue volumes with sub-cellular resolution throughout. *eLife* **5**:e18659. doi: 10.7554/eLife.18659

The idea of a microscope typically conjures up thoughts of a highly magnified image of a tiny sample captured in unprecedented detail. In principle, however, the physical mechanisms that allow us to see such microscopic details do not actually require the observed region or the specimen itself to be particularly small. Rather, the performance of a microscope is primarily limited by the diffraction of light as it passes through the various optical elements in the instrument.

The resolution of a microscope, d, is defined as the shortest distance between two points on a specimen that can still be seen as two distinct points in the image. In 1873 Ernst Abbe showed that diffraction limited the resolution of a microscope to $d=\frac{\lambda }{2n\;sin\;\alpha }$, where λ is the wavelength of the light, n is the refractive index of the medium the light travels through, and α is the half-angle at which the objective captures light from the sample: nsinα is also known as the "numerical aperture" of the objective.

As Abbe's equation makes clear, the resolution is independent of the magnification. Rather, it is the design of the objective that determines both the resolution of the microscope and its "field-of-view" (that is, the overall size of the sample that can be imaged). And although a variety of microscope objectives are available from commercial manufacturers, they are designed to either capture a fairly large field-of-view (typically a millimeter or so across) at low resolution, or a small field-of-view (typically a few hundred micrometers or less across) at high resolution. By contrast, to obtain a high-resolution image of a large specimen we need a combination of a large field-of-view, a long working distance (that is, the distance between the objective and the focal plane of the microscope), and a high numerical aperture: however, there are no commercial microscopes that offer this combination at present.

Now, in eLife, Gail McConnell of Strathclyde University and colleagues – Johanna Trägårdh, Rumelo Amor, John Dempster, Es Reid and Brad Amos (who is also at the MRC Laboratory for Molecular Biology) – report that they have developed an objective called the Mesolens that offers both a relatively large field-of-view and a relatively high resolution (Figure 1; McConnell et al., 2016). Using a custom-built confocal microscope equipped with the Mesolens, McConnell et al. were able to capture beautiful single-shot images of whole mouse embryos with cellular resolution, and also capture images of explants from the brains of rat embryos, again with cellular resolution.

**Figure 1.**
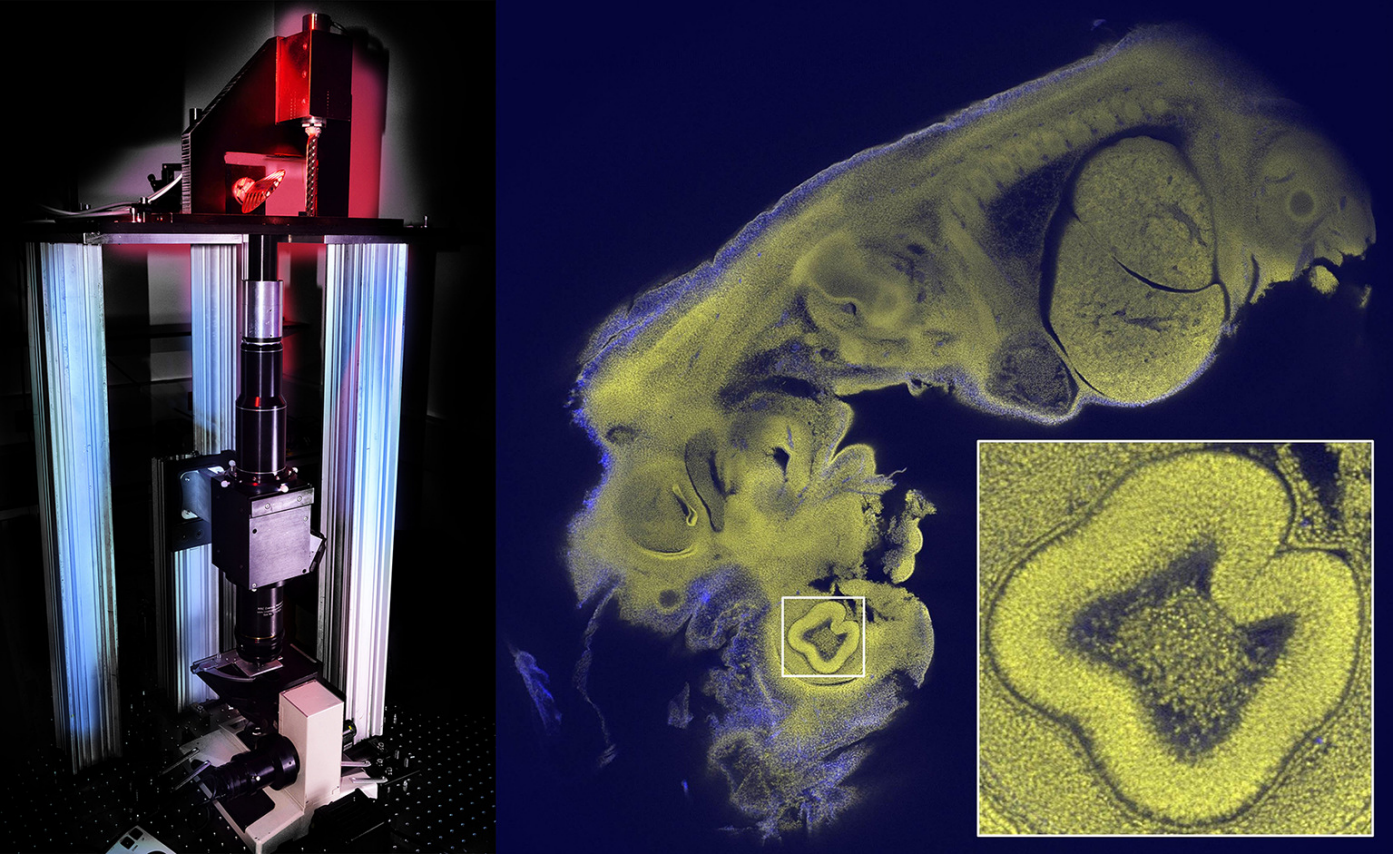
Imaging large samples with cellular resolution. The Mesolens microscope (left) contains a scanning system with two large beryllium mirrors (top; the mirror on the right can be seen side-on), a scan lens (black and silver cylinders), the Mesolens (two black cylinders, black cube and lower black cylinder), and a stage system to translate and focus the specimen (at the base). The Mesolens (which is 550 mm in length) is an immersion lens, and matching the immersion medium to the optical properties of the specimen greatly reduces spherical aberration, which is a common problem in light microscopy. The Mesolens design also incorporates corrections for both flat-field and chromatic aberration over a range of wavelengths. Image of a 12.5 day old mouse embryo (right); the field-of-view is 5 mm for the main image, and 0.46 mm for the inset; see Figure 5 of McConnell et al. for more details. Images courtesy of David Blatchford (left) and Johanna Trägårdh (right).

The Mesolens was made possible by a combination of sophisticated optical design and skillful engineering, including excellent correction for the various optical aberrations that would otherwise compromise high-resolution imaging over a large field-of-view. Moreover, the Mesolens is an immersion lens, which means that the gap between the objective and the sample is filled with a medium such as oil, water or glycerol: McConnell et al. designed their lens to provide high-quality images in a variety of immersion media with different optical properties.

A confocal microscope is able to image a sample in all three dimensions by taking two-dimensional images at a range of different depths: the Mesolens allowed McConnell et al. to image volumes of up to 6 x 6 x 3 mm^3^ with a lateral resolution of 0.7–0.8 micrometers and an axial resolution of 7–8 micrometers (with a numerical aperture of 0.47). These figures are factors of at least two and three higher than the resolutions that can be achieved with currently available large field-of-view commercial objectives.

In addition to applications in developmental biology and embryology, including whole-embryo phenotyping and screening applications that require rapid imaging of large specimens, the ability to image samples measuring several millimeters across with near-cellular resolution will be useful in many other areas of the life sciences. In neuroscience, for example, researchers are trying to combine techniques such as tissue clearing and tissue expansion (Höckendorf et al., 2014; Marx, 2016) with large-volume light microscopy (Ji et al., 2016) to image entire mammalian brains at high resolution; the Mesolens could prove useful in these efforts. And extending beyond its application in confocal microscopy, the Mesolens could have applications in light-sheet microscopy (Keller and Ahrens, 2015), which excels in the rapid imaging of large volumes. In particular, combining the Mesolens with other techniques could reduce the need to use "optical tiling" when imaging large specimens, or to subject large specimens to sectioning and other destructive sample-preparation techniques.

The Mesolens highlights the increasingly important role of custom optical designs in improving the performance of light-based microscopes. Recent, complementary efforts in mesoscale imaging with two-photon microscopy also rely heavily on custom-built objectives. For example, the two-photon random access microscope developed by Karel Svoboda of the HHMI Janelia Research Campus and co-workers features a custom objective with a numerical aperture of 0.6 and a field-of-view of 5 millimeters, and enables imaging up to a depth of 1 millimeter (Sofroniew et al., 2016). The twin-region, panoramic two-photon microscope (Trepan2p) developed by Spencer Smith of the University of North Carolina and co-workers features a custom objective with a numerical aperture of 0.43 and a field of view of 3.5 millimeters, and has been used to image multiple cortical areas in mice (Stirman et al., 2016). Other examples of this emerging trend to develop new microscopes with custom-built optics include lattice light-sheet microscopy (Chen et al., 2014) and IsoView light-sheet microscopy (Chhetri et al., 2015).

By extending the scale of optical microscopy to several millimeters, this new generation of custom-built objectives also emphasizes the need for new wide-field detection systems that can take full advantage of the capabilities of these new lenses. To optimally sample the large field-of-view captured by the Mesolens in a wide-field microscope, gigapixel cameras will be needed: for comparison, a state-of-the-art scientific camera will typically have 4–6 megapixels. Various gigapixel designs are already under development (Brady et al., 2012; Zheng et al., 2014), and further advances in this domain will help exploit the full potential of the Mesolens in imaging large volumes at high resolution.

## References

[bib1] Brady DJ, Gehm ME, Stack RA, Marks DL, Kittle DS, Golish DR, Vera EM, Feller SD (2012). Multiscale gigapixel photography. Nature.

[bib2] Chen BC, Legant WR, Wang K, Shao L, Milkie DE, Davidson MW, Janetopoulos C, Wu XS, Hammer JA, Liu Z, English BP, Mimori-Kiyosue Y, Romero DP, Ritter AT, Lippincott-Schwartz J, Fritz-Laylin L, Mullins RD, Mitchell DM, Bembenek JN, Reymann AC, Böhme R, Grill SW, Wang JT, Seydoux G, Tulu US, Kiehart DP, Betzig E (2014). Lattice light-sheet microscopy: Imaging molecules to embryos at high spatiotemporal resolution. Science.

[bib3] Chhetri RK, Amat F, Wan Y, Höckendorf B, Lemon WC, Keller PJ (2015). Whole-animal functional and developmental imaging with isotropic spatial resolution. Nature Methods.

[bib4] Höckendorf B, Lavis LD, Keller PJ (2014). Making biology transparent. Nature Biotechnology.

[bib5] Ji N, Freeman J, Smith SL (2016). Technologies for imaging neural activity in large volumes. Nature Neuroscience.

[bib6] Keller PJ, Ahrens MB (2015). Visualizing whole-brain activity and development at the single-cell level using light-sheet microscopy. Neuron.

[bib7] Marx V (2016). Optimizing probes to image cleared tissue. Nature Methods.

[bib8] McConnell G, Trägårdh J, Amor R, Dempster J, Reid E, Amos W (2016). A novel optical microscope for imaging large embryos and tissue volumes with sub-cellular resolution throughout. eLife.

[bib9] Sofroniew NJ, Flickinger D, King J, Svoboda K (2016). A large field of view two-photon mesoscope with subcellular resolution for in vivo imaging. eLife.

[bib10] Stirman JN, Smith IT, Kudenov MW, Smith SL (2016). Wide field-of-view, multi-region, two-photon imaging of neuronal activity in the mammalian brain. Nature Biotechnology.

[bib11] Zheng G, Ou X, Yang C (2014). 0.5 gigapixel microscopy using a flatbed scanner. Biomedical Optics Express.

